# Deletion of PPARα in mouse brown adipocytes increases their De Novo Lipogenesis

**DOI:** 10.1016/j.molmet.2025.102184

**Published:** 2025-06-10

**Authors:** Pierre-Louis Batrow, Sylvie Caspar-Bauguil, Nathalie Rochet, Nadine Gautier, Anne-Sophie Rousseau, Marielle Maret, Samah Rekima, Etienne Mouisel, Emmanuel Van Obberghen, Christian H. Roux, Hervé Guillou, Catherine Postic, Christian Wolfrum, Dominique Langin, Ez-Zoubir Amri, Isabelle Mothe-Satney

**Affiliations:** 1 Université Côte d’Azur, CNRS, Inserm, Adipocible Research Study Group, Institut de Biologie Valrose (iBV), Nice, France; 2 Institute of Metabolic and Cardiovascular Diseases, I2MC, University of Toulouse, Inserm, Toulouse III University - Paul Sabatier (UPS), Toulouse, France; 3 Université Côte d’Azur, CNRS, Inserm, IRCAN, Nice, France; 4 Université Côte d’Azur, CNRS, Laboratoire de PhysioMédecine Moléculaire (LP2M), Laboratories of Excellence Ion Channel Science and Therapeutics, Nice, France; 5 Rheumatology Department, Hospital Pasteur 2 CHU, Adipocible Research Study Group, 06000, Nice, France; 6 Toxalim (Research Centre in Food Toxicology), Université de Toulouse, INRAE, ENVT, INP-Purpan, UPS, Toulouse, France; 7 Université Paris Cité, Institut Cochin, CNRS, Inserm, Paris, France; 8 Laboratory of Translational Nutrition Biology, Institute of Food, Nutrition and Health, ETH Zurich, Schwerzenbach, Switzerland; 9 Department of Medical Biochemistry, Toulouse University Hospitals, France; 10 Institut Universitaire de France (IUF), Paris, France

**Keywords:** Peroxisome proliferator-activated receptor alpha, Brown adipose tissue, Inducible UCP1-CRE, High fat diet-induced obesity, β_3_-adrenergic stimulation, Lipid metabolism

## Abstract

**Objective:**

Peroxisome Proliferator-Activated Receptors (PPARs) are nuclear receptors involved in the control of lipid metabolism. The PPARα isoform is highly expressed in brown adipose tissue (BAT). However, its precise role in BAT remains unclear. Here, we aimed to investigate the role of PPARα in BAT of high fat diet-induced obese mice in a thermoneutral environment.

**Methods:**

We used tamoxifen-inducible-BAT specific PPARα knockout mice (PPARαBATKO) that were housed at thermoneutrality to minimize BAT basal activation, fed a high-fat diet for 20 weeks and challenged with a β_3_-adrenergic agonist (CL316,243) during the last week. Both male and female mice were studied.

**Results:**

Body weight and glucose tolerance tests were similar in both sexes and genotypes. However, BAT morphology was altered in PPARαBATKO mice, with more unilocular and larger lipid droplets compared to control mice, suggesting BAT impaired function. Indeed, when treated with CL316,243, both male and female mice had increased De Novo Lipogenesis (DNL), reflected by an increased expression of ChREBPβ and lipogenic enzymes ACLY, ACC1, FASN and SCD1. These changes were accompanied by an increase in fatty acids in triglycerides, and thus an increase in lipid storage. Moreover, lipid profiles in phospholipids were different, suggesting a modification in the membrane content with an increase of palmitoleate.

**Conclusions:**

Altogether, our results reveal a key role for PPARα in DNL in BAT and in the regulation of lipid metabolism in HFD-induced obesity.

## Highlights

• The lack of PPARα in BAT of HFD-induced obese mice maintained at thermoneutrality:
• Increases lipid droplet size in BAT in female mice.
• Induces BAT De Novo Lipogenesis (DNL) after β_3_-adrenergic stimulation in male and female mice.
• Increases fatty acid storage in triglycerides after β_3_-adrenergic stimulation in male and female mice.
• Increases palmitoleate in phospholipids and triglycerides after β_3_-adrenergic stimulation in male and female mice.

## Graphical abstract

**Figure:**
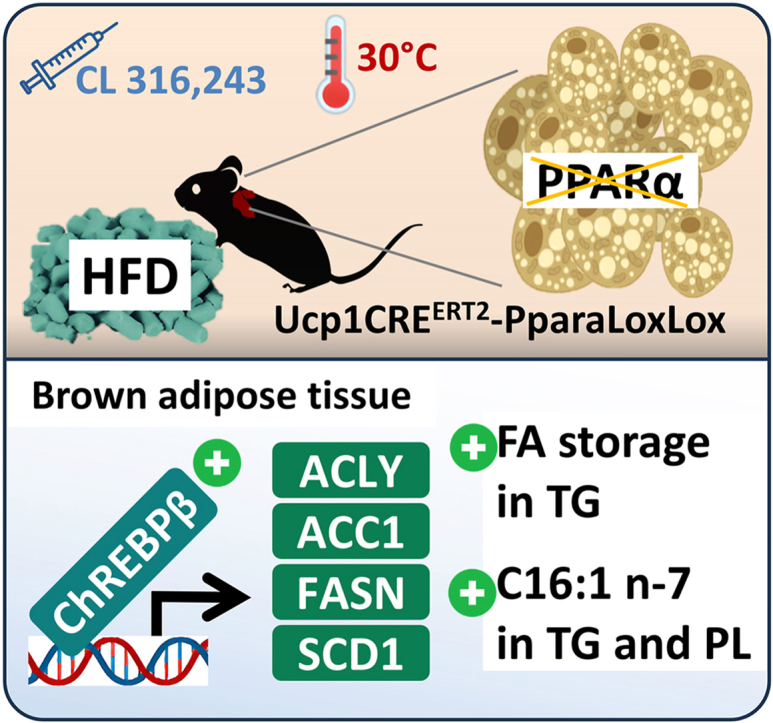

## 1 Introduction

The prevalence of overweight and obesity is increasing worldwide reaching epidemic proportions and is attributed to multiple factors, including changes in lifestyle, increased consumption of calorie rich food and insufficient physical activity [1,2]. Targeting fat cell metabolism to increase energy expenditure is investigated as a promising path to counter obesity. One way to increase energy expenditure is through non-shivering thermogenesis in brown adipose tissue (BAT). Positron Emission Tomography (PET)-scan analyses show that healthy human adults possess active BAT depots [[3], [4], [5]]. Non-shivering thermogenesis is achieved mainly through the activation of uncoupling protein 1 (UCP1), which is highly expressed in BAT [6]. This protein is induced by specific stimuli, such as cold exposure in BAT, but also in WAT, giving rise to beige or brite adipocytes [7,8]. Importantly, the peroxisome proliferator-activated receptor (PPAR) family has been implicated in adipocyte browning [7,9]. It consists of three different isoforms (α, β/δ, γ) involved in fatty acid metabolism [[10], [11], [12]]. PPARδ is the most ubiquitous of the three isoforms and plays a key role in skeletal muscle. Studies on mice lacking PPARδ specifically in BAT showed that PPARδ was dispensable for BAT function [13]. PPARγ expression is important in adipose tissue and is critical for both adipogenesis and thermogenesis [13]. Global deletion of PPARγ results in embryonic death at E10 [14]. Inactivating mutations of PPARγ cause lipodystrophy and increased risk of type 2 diabetes (T2D) in humans [15]. Synthetic selective PPARγ activators, including thiazolidinediones (TZDs) such as rosiglitazone, have been shown to induce the thermogenic program in mouse WAT [7]. Rosiglitazone also promotes the conversion of human white adipocytes into beige adipocytes *in vitro* [16]. PPARα is present in tissues with high oxidative capacity, particularly BAT and liver, and is considered as a major regulator of hepatic lipid metabolism [17,18]. The role of PPARα in adipose tissue remains unclear even if several studies have been reported [13,[19], [20], [21]]. PPARα regulates SREBP-mediated adipogenesis in WAT. Its expression is induced in this tissue upon cold exposure [19,22] and rapidly decreases after removal of thermogenic stimulation such as treatment with rosiglitazone or β3-adrenergic agonist [23]. Cold exposure induces transient hypothermia in PPARα−/− mice [19]. However, whole genome expression profiling of the inguinal WAT depot showed no difference between wildtype and PPARα−/− mice exposed to thermoneutrality or cold for ten days [19]. Mice lacking PPARα in cells expressing adiponectin or UCP1 display unaltered UCP1 levels and normal cold tolerance [13,21]. These studies were carried out with mice fed a standard chow diet. *In vivo* and *in vitro* studies indicated that dual PPARα/γ activation synergistically favored UCP1-dependent and independent thermogenesis [20,24]. Specific PPARα activators such as pemafibrate can induce browning of white adipocytes, but to a lesser extent than rosiglitazone [23,25]. Moreover, effects of pemafibrate were more potent *in vivo* than *in vitro* and were predominantly mediated by increases in Fibroblast Growth Factor 21 (FGF21) expression in the liver [23,26,27]. The importance of PPARα in BAT therefore remains unclear, and its role in BAT of high-fat diet (HFD)-induced obese mice has not yet been investigated. In the present study, we studied the specific role of PPARα in BAT using an *in vivo* knockout mice model. In brief, female and male mice lacking PPARα specifically in brown adipocytes were housed at thermoneutrality and fed a HFD. Treatment with the β3-adrenergic agonist CL316,243 suggests that PPARα might regulate BAT thermogenic capacity only in female mice. More importantly, the absence of PPARα leads to increased De Novo Lipogenesis (DNL) in both sexes, indicating a newly identified role for PPARα in BAT.

## 2 Materials and methods

### 2.1 Animals and housing

The experiments were conducted in accordance with the French and European regulations (directive 2010/63/EU) for the care and use of research animals and were approved by national experimentation committees. Male and female mice of the inbred strain C57BL/6N were bred and housed at room temperature (22 ± 2 °C) with 12:12 h light–dark cycles and fed chow diet (CD) with ad libitum access to food and water prior to the beginning of experiments. At the age of 7–9 weeks, mice were transferred to ventilated cabinets at thermoneutrality (28–30 °C).

The mouse line UCP1-CRE^ERT2^ was obtained from C. Wolfrum’s lab (ETH Zurich, Switzerland). This mouse was extensively phenotyped and no phenotypic alterations due to the presence of CRE^ERT2^ were observed [28]. UCP1-CRE^ERT2^-PPARalpha^lox/lox^ (PPARαBATKO) and UCP1-WT-PPARalpha^lox/lox^ (Ctrl) mice were described by Lasar et al. [13]. At the age of 15–16 weeks, PPARαBATKO and Ctrl littermates were divided in 2 groups, fed for 20 weeks either a Chow Diet CD (SAFE A03) or a High-Fat Diet HFD (SNIFF reference #E15742-347, with 60% energy content as lipids from lard). To induce CRE^ERT2^ activity, Tamoxifen (T5648-5G) was dissolved in sunflower seed oil (Sigma, S5007) at a concentration of 15 mg/mL by incubating at 42 °C overnight. The mice received intraperitoneal injections of tamoxifen at a dosage of 60 mg/kg the first three days of the experiment (beginning of the 20-week diet), then every three weeks in order to target newly formed adipocytes. After 13 weeks of diet, glucose tolerance was assessed. Briefly, after 6 h of fasting, mice received an intraperitoneal injection of glucose (2 g/kg body weight). Blood glucose levels were measured at incised tail tips before (0 min) and at 15, 30, 60, 90 and 120 min after glucose injection. In order to activate BAT thermogenesis, β3-adrenergic receptor stimulation was carried out during the last week of the diet treatment by daily intra-peritoneal injections of CL316,243 (1 mg/kg in saline solution) at the same time of the day. Ctrl mice were injected with vehicle only. One day after the last CL316,243 injection, mice were anesthetized and subjected to tomography. Then, blood was recovered through heart punction and mice were euthanized (cervical dislocation). Blood, interscapular BAT (BAT), epididymal WAT (eWAT), inguinal subcutaneous WAT (scWAT) and liver were sampled and used for different analyses.

### 2.2 Tomography

Adipose tissue quantification was carried out using a SkyScan1178 X-ray micro-CT system as previously described [29]. Briefly, mice were anesthetized and scanned using the following parameters: 104 μm of voxel size, 49 kV, 0.5 mm thick aluminum filter, 0.9° of rotation step. Adipose tissue volume (AT volume) was measured between caudal vertebra 1 (C1) and thoracic vertebra 13 (T13). 3D reconstructions and analysis of AT volumes were performed using NRecon and CTAn software (Skyscan).

### 2.3 Histology and measurement of lipid droplet size

Inguinal WAT, interscapular BAT and liver were fixed in 4% (V/V) paraformaldehyde for 48 h, then stored in 0.4% (V/V) paraformaldehyde until processed. Following dehydration and paraffin-embedding, 5 μm sections were stained with hematoxylin and eosin, or with Picro-Sirius red to evaluate fibrosis. Quantification of fibrosis was performed using ImageJ software. Sections were mounted, dried and analyzed by bright field microscopy (Slide Scanner Vectra Polaris). Displayed images are representative of four mice analyzed per group. Images of individual BAT tissues were extracted at a 0.249 μm resolution using QuPath [30]. Lipid Droplet (LD) detection, measurement and distribution were performed using a collection of Python Jupyter Notebooks. The images were opened (https://github.com/AllenCellModeling/aicsimageio), converted in grayscale and inverted before inference with the Cellpose [31] algorithm at two different scales (2 μm/8pixels and 13,68 μm/54.9 pixels) with a model trained for LD segmentation for each scale. Each model was trained with transfer learning from the Cellpose’s cyto2 model with the rescale images during training option on false and on a dataset manually resized to the respective inference scale. Datasets contains 2 images for scale 2 μm and 16 images for scale 13,68 μm and were annotated with the ImageJ software. The label images resulting from the inference are filtered with a size bandwidth adapted to each scale. Then these different scales are merged by adding them successively in increasing order. Labels were added if less than 10% of their area is occupied by other labels from smaller scales. On the resulting multi-scale, image labels area are measured, then converted to an equivalent diameter.

### 2.4 Isolation and analysis of RNA

These procedures followed Minimum Information for Publication of Quantitative Real-Time PCR Experiments (MIQE) standard recommendations and were conducted as described previously [32,33]. Total RNA was extracted using TRI-Reagent kit (Euromedex, Souffelweyersheim, France) according to the manufacturer’s instructions. RT-PCR was performed on 1 μg of RNA using M-MLV-RT (Promega). Quantitative PCR (qPCR) was performed using ONEGreen® Fast qPCR Premix (Ozyme, Montigny-le-Bretonneux, France), and assays were run on a StepOne Plus ABI real-time PCR machine (PerkinElmer Life and Analytical Sciences, Boston, USA). The expression of selected genes was normalized to that of the 36B4 housekeeping gene and then quantified using the comparative-ΔCt method. The oligonucleotide sequences are shown in Supplementary Table 1.

### 2.5 Western blot analysis

Interscapular BAT was fine powdered in liquid nitrogen, solubilized in lysis buffer followed by sonication as previously described [33]. After centrifugation performed at 14,000 RPM for 10 min, protein content was quantified with BCA kit. Equal amounts of total proteins, 10–30 μg, were separated by 10 or 12% SDS-PAGE gel electrophoresis and transferred onto the PVDF membrane. Following blocking, membranes were incubated with primary antibody overnight at 4 °C (Supplementary Table 2). Primary antibodies were detected with HRP-conjugated anti-rabbit or anti-mouse immunoglobulins (Promega, Charbonnieres Les Bains, France). Chemiluminescence obtained after adding Immobilon Forte Western HRP substrate (Merck-Millipore, ST Quentin En Yvelines, France) was detected using an Amersham Imager 600. Loading control was performed with total Coomassie blue staining, pan-actin or tubulin content.

### 2.6 Isolation and analysis of DNA

Genomic and mitochondrial DNA were extracted from iBAT by the PureLink Genomic DNA kits (Invitrogen, Villebon Sur Yvette, France). Quantitative PCR was performed on 3 ng of total DNA using Cox1 primers for mitochondrial DNA and Ppia primers for nuclear DNA. Mitochondrial DNA was calculated as 2ΔCt of Cox1 with Ppia as housekeeping gene. The oligonucleotide sequences are shown in Supplementary Table 1.

### 2.7 Plasma analyses

Blood was collected in heparinized tubes via cardiac puncture. Plasma was obtained after 10 min of centrifugation at 4000 rpm and stored at −80 °C until further analyses. Adiponectin, leptin and FGF21 levels were measured using Adiponectin (mouse) ELISA (Bertin Bioreagent, A05187), Leptin (mouse, rat) ELISA (Bertin Bioreagent, A05176) and Fibroblast Growth Factor 21 Mouse/Rat ELISA (Biovendor, RD291108200R) specific kits, respectively.

### 2.8 Lipidomics

Fatty acid (FA) composition of BAT triglycerides was determined by capillary gas chromatography. Tissue samples were homogenized in methanol/butylated hydroxy toluene (10 mg/L) and stored at −20 °C until analysis. Neutral lipids (corresponding to an equivalent of 20 mg tissue) were extracted following the Folch method using chloroform/methanol (2:1 vol/vol), in the presence of the internal standard glyceryl trinonadecanoate (Sigma–Aldrich). The extracts were filtered, and lipids recovered in the methanol-chloroform phase. Triglycerides (TG) and Phospholipids (PL) were isolated using thin layer chromatography on silica glass plates (E. Merck, Darmstadt, Germany) developed in petroleum ether, ethyl ether, acetic acid (80:20:1) and visualized by fluorescein (2,7 – dichlorofluoresceine (Sigma) 0.2% in ethanol). The TG and PL bands were scraped from the plate and transmethylated using 5% acetyl chloride/95 % methanol. The methylated fatty acids were extracted with isooctane and analyzed by gas chromatography using a gas chromatograph GC30 equipped with flame ionization detectors (Shimatzu), and CP-Wax 58 capillary column (50 mm in length, 0.25 mm external diameter, 0.2 μm thickness of the stationary phase (Varian Inc., Les Ulis, France)). Helium was used as a carrier gas. Fatty acid methyl esters are identified by comparing the retention times to those of known standards.

### 2.9 Quantification and statistical analysis

Data are expressed as mean values ± SEM and were analyzed using Prism 9.1.1 software (GraphPad Software). Shapiro–Wilk test was used to test distributions normality. For normally distributed variables, data were analyzed by two-way ANOVA followed by Šídák’s post hoc test to assess statistical differences between experimental groups. Differences between two groups were analyzed using unpaired two-tailed Student’s t-test. For non-normally distributed variables, data were analyzed by Mann–Whitney test. Differences were considered statistically significant with *p* < 0.05.

## 3 Results

### 3.1 Generation of PPARαBATKO mice by inducible deletion of PPARα specifically in BAT

To investigate the role of PPARα in mature brown adipocytes, we generated PPARαBATKO mice by crossing PPARα^lox/lox^ mice with UCP1-CRE^ERT2^ mice (Figure 1A) [13]. The resulting mice and control littermates (Ctrl) were maintained at thermoneutrality (28–30 °C) and were given a HFD for 20 weeks. The last 7 days of the experiment, mice of each genotype were divided into 2 groups (Figure 1B). Half of the animals were daily injected with 1 mg/kg of CL316,243, while the other half was injected with vehicle (NaCl). PPARαBATKO mice displayed, upon tamoxifen treatment, a strong inhibition of Pparα mRNA levels (Figure 1C) in BAT compared to Ctrl mice (up to 97% in female and 83% in male mice), whereas Pparγ and Pparδ levels were not affected (Supplementary Figure 1A and C). β3-adrenergic agonist stimulation had no significant effect on Pparα expression in BAT. Female PPARαBATKO mice displayed a slight decrease in Pparα mRNA level in scWAT in NaCl condition (Figure 1D), while CL316,243 had no significant effect on Pparα expression in both genotypes of male and female mice. The lack of Pparα expression in BAT did not alter its level in liver of both female and male mice (Figure 1E). It is noteworthy that while Pparα mRNA levels are high in the liver in NaCl condition, they are also relatively high in the BAT, whereas they are much lower in scWAT (Figure 1C–E). Circulating levels of FGF21 were the same in both genotypes (Supplementary Figure 1B). Together our data showed that PPARαBATKO is an appropriate male or female model to investigate the role of PPARα specifically in BAT.

**Figure 1:**
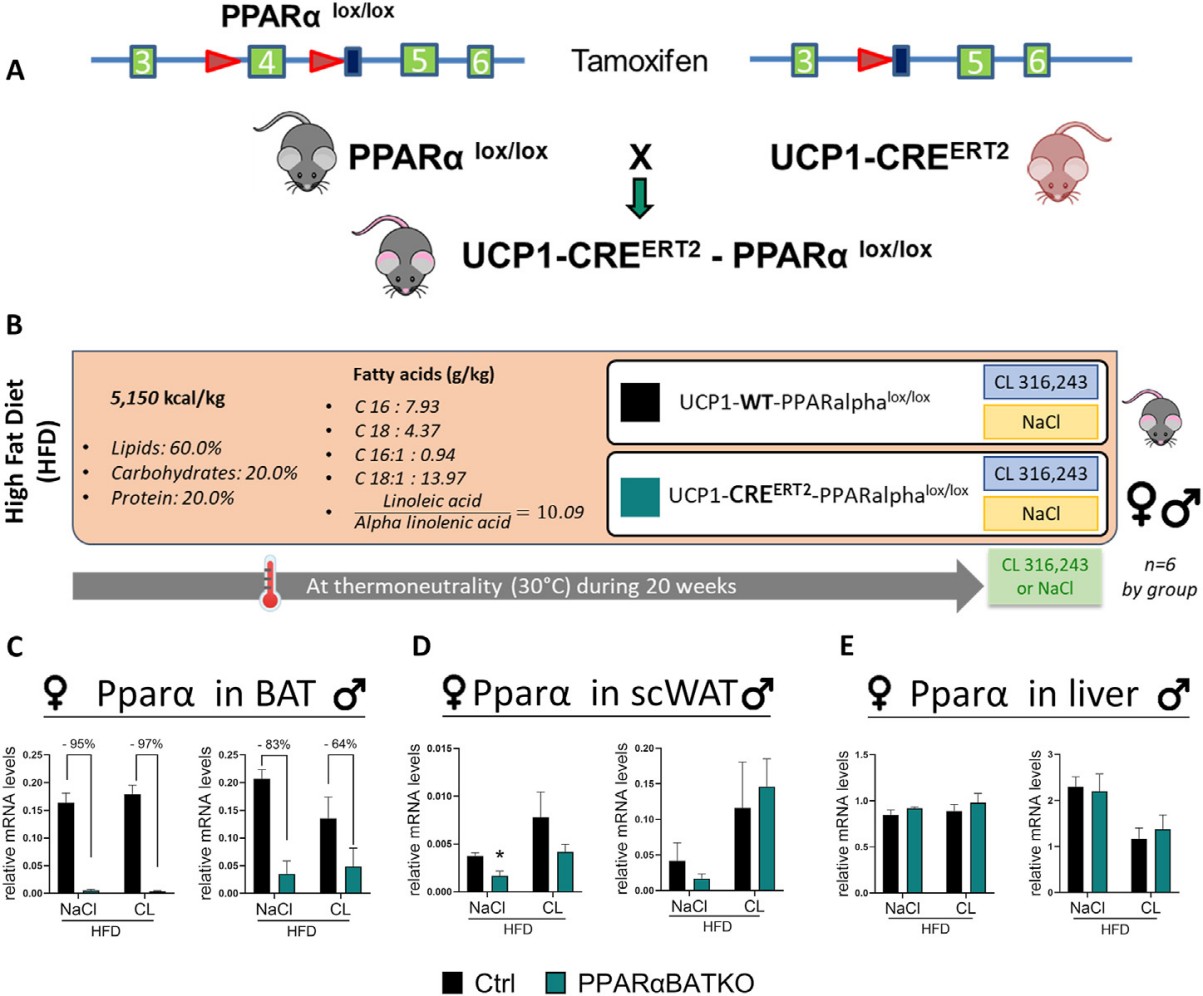
**Generation of PPARαBATKO mice showing deletion of PPARα specifically in brown adipose tissue.** (A) Schematic representation for the generation of PPARαBATKO. (B) Experimental design. Control (UCP1-WT-PPARalphalox/lox) and PPARαBATKO (UCP1-CRE^ERT2^-PPARalphalox/lox) were housed at thermoneutrality and fed a high fat diet (HFD) for 20 weeks and daily injected with 1 mg/kg of CL316,243 or NaCl the last week of the experiment. To induce CRE^ERT2^ activity, mice were injected intraperitoneally with tamoxifen at a dosage of 60 mg/kg the first three days of the HFD, then every three weeks. (C) Relative mRNA levels of Pparα in brown adipose tissue (BAT) of female and male adult mice (*n* = 5–6 per group). (D) Relative mRNA levels of Pparα in subcutaneous (scWAT) of female and male mice (*n* = 5–6 per group). (E) Relative mRNA levels of Pparα in liver of female and male mice (*n* = 3–6 per group). Data are displayed as mean ± SEM. Statistical analysis was performed using 2-way ANOVA with Šídák’s post hoc test (C, E for female) or Mann–Whitney test (D, E for male); ∗*p* < 0.05, *vs* Ctrl.

### 3.2 BAT specific deletion of PPARα does not alter glucose tolerance or body weight gain in HFD-induced obese mice

PPARαBATKO mice displayed a similar body weight compared to Ctrl mice during the 20 weeks of HFD (Figure 2A,H). The absence of PPARα in BAT did not alter glucose tolerance, neither in female nor in male mice (Figure 2B,I). The last week of the experiment consisted for half of the animals in BAT stimulation with daily β3-adrenergic agonist injections (CL316,243). In female mice, no significant difference was observed between both genotypes for total body weight (Figure 2C), fat pads weight of interscapular BAT or scWAT (Figure 2D). The body weight of PPARαBATKO male mice treated with vehicle was comparable to that of Ctrl mice (Figure 2J). However, when treated with β3-adrenergic agonist, total body weight was significantly higher in male PPARαBATKO mice (Figure 2J), reaching the weight of mice treated with NaCl. In male mice, CL316,243 treatment significantly reduced BAT weight, but only in Ctrl mice, suggesting the occurrence of an altered response to β3-adrenergic stimulation in PPARαBATKO male mice (Figure 2K). As observed in female mice, the weight of scWAT was the same between both genotypes in male mice treated with vehicle or CL316,243. In female mice, AT volume, estimated by tomography, was significantly reduced by β3-adrenergic agonist treatment only in Ctrl mice (Figure 2E). In male mice, AT volume was slightly reduced by CL316,243 in Ctrl mice, while it was not modified in PPARαBATKO male mice (Figure 2L).

**Figure 2:**
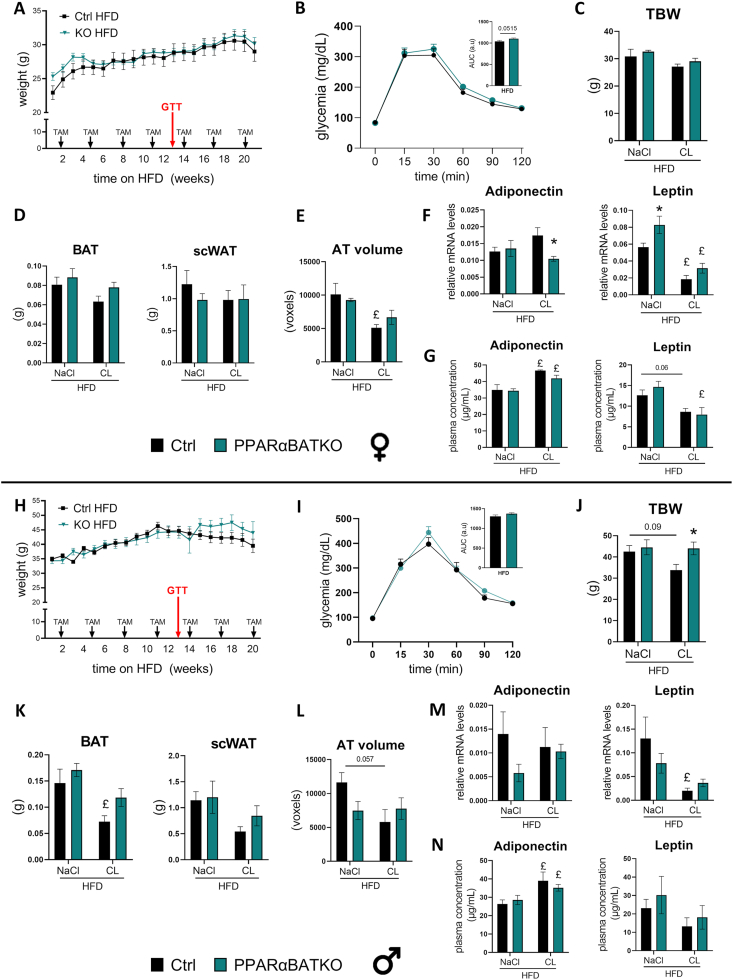
BAT specific deletion of PPARα does not alter glucose tolerance or body weight gain in HFD-induced obese mice. (A, H) Weight curves of control (Ctrl) and PPARαBATKO female (A) and male (H) mice (*n* = 12 per group) on high fat diet (HFD) during 20 weeks. To induce CRE^ERT2^ activity, mice were injected intraperitoneally with tamoxifen (TAM) at a dosage of 60 mg/kg the first three days of the HFD, then every three weeks. (B, I) Glucose tolerance test (GTT) after 13 weeks of HFD in female (B) and male (I) mice, and corresponding area under curves (AUC) (*n* = 8–12 per group). (C, J) Total Body weight (TBW) in female (E) and male (G) mice (*n* = 4–6 per group). (D, K) Interscapular brown adipose tissue (BAT) and subcutaneous white adipose tissue (scWAT) weights in female (D) and male (K) mice (*n* = 4 = 6 per group). (E, L) AT volume estimation assessed by tomography in female (E) and male (L) adult mice (*n* = 3–6 per group). (F, M) Relative mRNA levels of Adiponectin and Leptin in BAT of female (F) and male (M) adult mice (*n* = 4–6 per group). (G, N) Plasma levels of Adiponectin and Leptin in female (G) and male (N) mice (*n* = 4–6 per group). Data are displayed as mean ± SEM. Statistical analysis was performed using 2-way ANOVA with Šídák’s post hoc test, Mann–Whitney test (E for TBW, J for Adiponectin) or student t-test for AUC. ∗*p* < 0.05, *vs* Ctrl. £ *p* < 0.05, *vs* NaCl. NaCl: vehicle; CL: CL316,243 (β3-adrenergic agonist).

Although the absence of PPARα in BAT did not modify glucose tolerance or body weight in female mice, PPARαBATKO female mice displayed changes in adipose tissue gene expression (Figure 2F). BAT adiponectin mRNA level after β3-adrenergic agonist stimulation was significantly lower in PPARαBATKO mice compared to Ctrl mice (Figure 2F). In female PPARαBATKO mice treated with vehicle, BAT leptin mRNA level was also significantly higher compared to Ctrl mice (Figure 2F). The amounts of these mRNAs in BAT were in agreement with the tendencies observed for the corresponding adipokine levels in plasma (Figure 2G). In male mice, mRNA level of adiponectin and corresponding circulating level were the same in both genotypes (Figure 2M,N). In male mice, β3-adrenergic agonist treatment induced a reduction of leptin mRNA only in Ctrl mice (Figure 2M). However, plasma level of leptin was the same in both genotypes (Figure 2N). Together, these data suggest that BAT specific deletion of PPARα does not alter glucose tolerance, but might interfere with the response induced by β3-adrenergic agonist in BAT.

### 3.3 BAT specific deletion of PPARα alters BAT morphology and slightly changes the β3-adrenergic agonist-induced BAT activation in HFD-induced obese female mice

To delve into the role of PPARα in BAT, we performed histological analyses. Hematoxylin and eosin staining revealed that the BAT of female PPARαBATKO mice exhibited more unilocular and larger lipid droplets (LDs) compared to Ctrl mice when treated with vehicle (Figure 3A,C). The distribution of LD size in BAT was slightly altered in PPARαBATKO mice in both the vehicle- and β3-adrenergic agonist-treated groups (Figure 3B). Indeed, the mean LD diameter was significantly higher in PPARαBATKO mice treated with vehicle (Figure 3C), due to a reduction in small LDs and a trend toward an increase in larger LDs (Figure 3D, left panel). β3-adrenergic agonist treatment reduced the mean LD size in both genotypes (Figure 3A,C). However, PPARαBATKO mice treated with CL exhibited a reduced proportion of very small LDs, along with an increased proportion of intermediate-sized LDs (Figure 3D, right panel). Morphological changes observed in BAT of female PPARαBATKO mice were not seen in all BATs of male PPARαBATKO mice and therefore were not statistically different from Ctrl mice (data not shown). As a morphological change in adipose tissue is often associated to extracellular matrix remodeling, we stained BAT sections with Picro-Sirius Red. After β3 adrenergic agonist stimulation, the collagen deposition area was increased in female PPARαBATKO mice, (Figure 3E). However, mRNA levels of Col1a1 and Col6a1 were similar in both genotypes (Figure 3F). Histological analyses showed that BAT specific deletion of PPARα altered brown adipocyte morphology, especially in female mice. As PPARα activation has been shown to be involved in WAT browning, we investigated the expression of genes involved in BAT thermogenesis. In female mice, the level of Ucp1 mRNA, the main gene involved in thermogenesis, was similar in BAT of both genotypes fed a HFD and treated with vehicle (Figure 3G). β3 adrenergic agonist stimulation induced Ucp1 expression in both genotypes. However, this effect was significantly reduced in female PPARαBATKO mice compared to Ctrl mice, at the mRNA level (Figure 3G), but was unchanged at the protein level (Figure 3H). This slight reduction in CL induced Ucp1 level was also observed in female mice fed a CD (Supplementary Figure 2A). In female mice fed a HFD, but not fed a CD, the expression of other thermogenesis-related genes was impaired in the absence of PPARα in BAT: Cpt1m and Ppargc1α mRNA levels were lower in female PPARαBATKO mice in both NaCl and CL conditions (Supplementary Figure 2B). BAT specific deletion of PPARα also impaired the induction of Atgl and Lpl, but only in female mice fed a HFD (Supplementary Figure 2B). We further investigated female mice fed a HFD. GK1 protein level was reduced in female mice PPAαRαBATKO compared to Ctrl mice (Supplementary 11 Figure 2C). Moreover, GK1 and DRP1 induction by CL316,243 was blunted in the absence of PPARα in BAT (Supplementary Figure 2C). As DRP1 is involved in mitochondrial fission and recycling, we then investigated the relative mitochondrial DNA content. When PPARα is absent in BAT, mitochondrial DNA content (calculated as 2ΔCt of Cox1 with Ppia as housekeeping gene) was decreased by 40% after β3-adrenergic agonist treatment compared to Ctrl in HFD-induced obese female mice (Supplementary Figure 2D). However, OXPHOS protein levels appeared to be similar in Ctrl and PPARαBATKO female mice (Supplementary Figure 2E). In male mice fed a HFD, the absence of PPARα in BAT did not alter the expression of genes related to thermogenesis or mitochondrial biogenesis (Supplementary Figure 3A). Relative mitochondrial DNA content was only slightly reduced in PPARαBATKO male mice treated with β3-adrenergic agonist (Supplementary Figure 3B). These data show that the absence of BAT PPARα in female mice fed a HFD induces a whitening of BAT in basal conditions and a lesser activation of BAT in response to the β3-adrenergic agonist, associated with an alteration of tissue morphology and mitochondrial gene expression.

**Figure 3:**
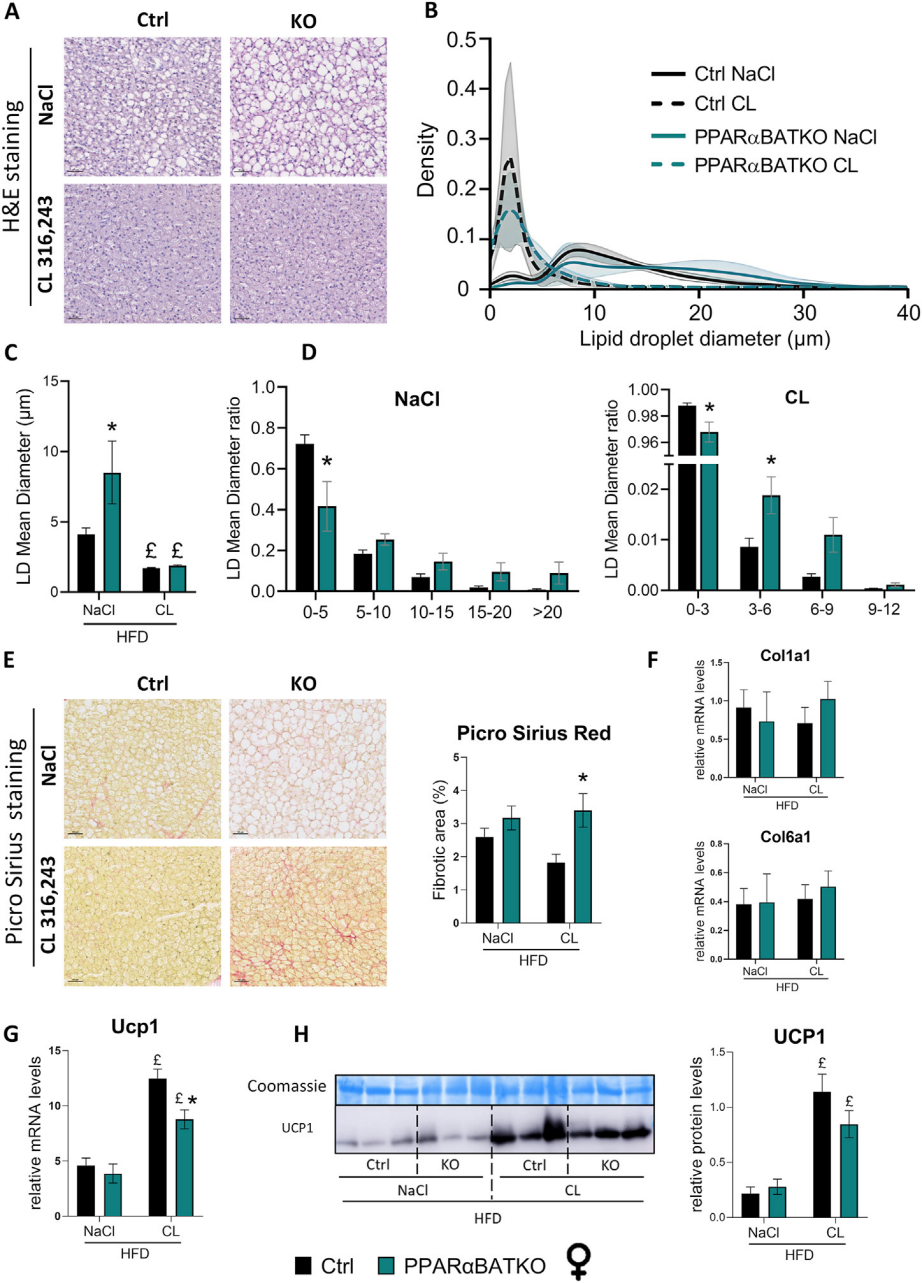
**BAT specific deletion of PPARα alters BAT morphology and slightly changes the β3-adrenergic agonist-induced BAT activation in HFD-induced obese female mice.** (A) Representative H&E staining of brown adipose tissue (BAT) in female mice fed a HFD treated with vehicle (NaCl) or β3-adrenergic agonist (scale = 40 μm). (B) Lipid droplet (LD) diameter distribution in female mice treated with vehicle (NaCl) or β3-adrenergic agonist (*n* = 4 per group). (C) LD average diameter (μm) in female mice treated with vehicle (NaCl) or β3-adrenergic agonist (*n* = 4 per group). (D) Class distribution of LD diameters (in μm) to the total number of lipid droplets in female mice fed a HFD and treated with NaCl (left panel) or β3-adrenergic agonist (right panel) (*n* = 4 per group). (E) Representative Picro-Sirius red staining of BAT in female mice (scale = 40 μm) and corresponding quantification. (F) Relative mRNA levels of Col1a1 and Col6a1 in BAT of female mice (*n* = 5–6 per group). (G) Relative mRNA levels of Ucp1 in (BAT) of female mice (*n* = 4–6 per group). (H) Western blot of UCP1 protein content in BAT of female mice and corresponding quantification (*n* = 3 per group). Data are displayed as mean ± SEM. Statistical analysis was performed using Mann–Whitney test (F) or 2-way ANOVA with Šídák’s post hoc test (E, G and H) or Fisher’s LSD test (C and D). ∗*p* < 0.05, vs Ctrl. £*p* < 0.05, vs NaCl. NaCl: vehicle; CL: CL316,243 (β3-adrenergic agonist). Ctrl: control mice; KO: PPARαBATKO mice.

### 3.4 BAT specific deletion of PPARα increases BAT De Novo Lipogenesis and fat storage in HFD-induced obese mice upon β3-adrenergic stimulation

DNL is an essential process for lipid deposition in both WAT and BAT. Obesogenic diets induce a reduction of DNL in both WAT and BAT [34]. Here, Ctrl female and male mice fed a HFD showed low expression levels of genes involved in the DNL pathway. Indeed, in control genotype, the mRNA levels of ChREBPβ and its target genes involved in DNL (Acly, Acaca and Fasn) were low and not modified after β3-adrenergic agonist treatment (Figure 4A,G). However, both female and male PPARαBATKO mice fed a HFD displayed a strong induction of ChREBPβ mRNA level after β3-adrenergic agonist treatment (Figure 4A,G). Chrebpa and Srebp1 levels were not modified in the absence of PPARα in BAT (Supplementary Figure 1D and 1E). Interestingly, ChREBP–regulated genes such as Acly, Acaca and Fasn were also highly induced in PPARαBATKO mice treated with β3-adrenergic agonist, but the expression of Acacb was the same in both genotypes. Western-blot analyses confirmed these inductions for ACLY, ACC and FASN at protein level with no modification of ACC phosphorylation (Figure 4B,C). We performed fatty acid (FA) profiling in BAT triglycerides (TG) and phospholipids (PL) using gas chromatography to explore the physiological consequences of increased expression of lipogenic genes in PPARαBATKO mice (Supplementary Figure 4A and 4B). Total FA content of TG in BAT was increased in male PPARαBATKO mice treated with β3-adrenergic agonist compared to Ctrl mice (Figure 4I). Molar percentage of C16:0 found in BAT TG tended to be increased in female PPARαBATKO mice treated with β3-adrenergic agonist (Figure 4D, left panel), reaching significant threshold in male mice (Figure 4H, left panel). Palmitate relative abundancy was also significantly higher in male PPARαBATKO mice treated with the vehicle (Figure 4H, left panel). However, the molar percentage of palmitate in BAT PL was the same in both genotypes (data not shown). The ratio of palmitate (C16:0) to linoleic acid (18:2 n-6) is a marker of the amount of endogenously synthesized versus dietary supplied FAs. This lipogenic index was increased in BAT TG of both female (Figure 4D, right panel) and male (Figure 4H, right panel) PPARαBATKO mice treated with CL316,243. It was also augmented with vehicle in male PPARαBATKO mice (Figure 4H, right panel). However, this FA ratio in BAT PL remained the same in both genotypes (data not shown). Since ChREBPβ is also known to control the expression of genes involved in glycolysis [35], we looked at the expression of PKM2, one of the rate-limiting enzymes in glycolysis [36]. Like ChREBP-DNL targets, PKM2 is up-regulated in PPARαBATKO mice upon β3-adrenergic agonist treatment (Figure 4E,J). Finally, we investigated AKT signaling as AKT2 was found to be a cold-induced-kinase involved in ChREBP-mediated DNL in BAT [34]. Interestingly, we found, at least in female PPARαBATKO mice treated with β3-adrenergic agonist, an increased expression of AKT2 protein (50%) associated to changes in DNL enzymes (Figure 4F). These data illustrate a role for PPARα in the regulation of DNL leading to lipid accumulation in TG which may be associated with a switch toward glycolytic metabolism after a double challenge consisting in HFD followed by β3-adrenergic stimulation.

**Figure 4:**
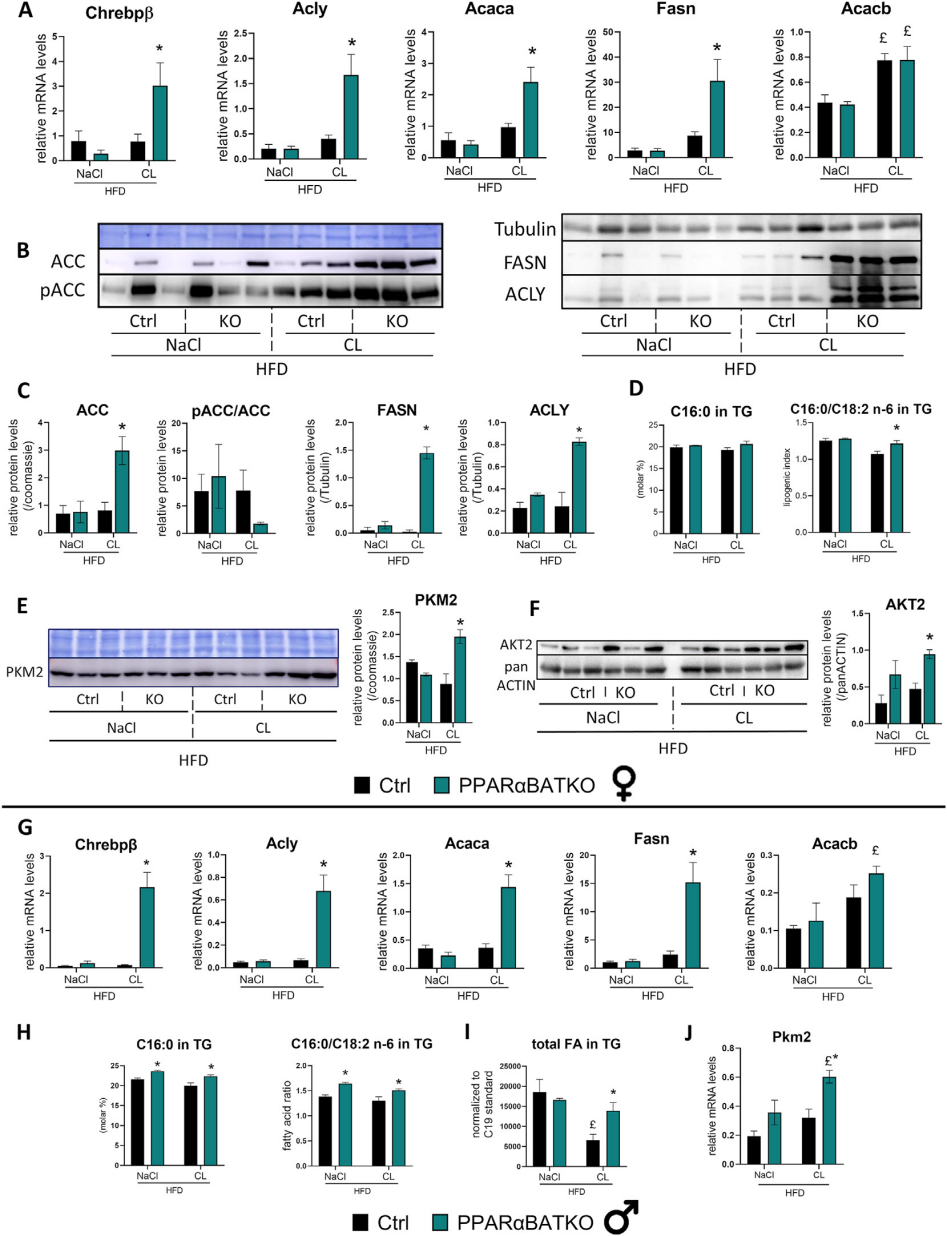
**BAT specific deletion of PPARα increases BAT De Novo Lipogenesis for fat storage in HFD-induced obese mice upon β3-adrenergic stimulation.** (A, G) Relative mRNA levels of De Novo Lipogenesis (DNL) genes in brown adipose tissue (BAT) of female (A) and male (G) mice (*n* = 4–6 per group). (B) Western blots of DNL proteins in BAT of female mice and corresponding quantifications (C) (*n* = 3 per group). (D, H) Relative abundancy of palmitate and lipogenic index in BAT triglycerides (TG) of female (D) and male (H) mice (*n* = 4 per group). (E) Western blot of PKM2 protein in BAT of female mice and corresponding quantification (*n* = 3 per group). (F) Western blot of AKT2 protein in BAT of female mice and corresponding quantification (*n* = 3 per group). (I) Relative fatty acid content in BAT triglycerides compared to internal standard C19 of male mice (*n* = 4 per group). (J) Relative mRNA levels of Pkm2 in BAT of male mice (*n* = 4–6 per group). Data are displayed as mean ± SEM. Statistical analysis was performed using 2-way ANOVA with Šídák’s post hoc test or Mann–Whitney test (A for Chrebpb, Acly, Fasn, and G, Chrebpb, Acly, Acaca, Fasn). ∗*p* < 0.05, *vs* Ctrl. £*p* < 0.05, *vs* NaCl. NaCl: vehicle; CL: CL316,243 (β3-adrenergic agonist). Ctrl: control mice; KO: PPARαBATKO mice.

### 3.5 BAT specific deletion of PPARα increases FA desaturation in BAT in HFD-induced obese mice upon β3-adrenergic stimulation

Next, we investigated whether PPARα regulated palmitate elongation and desaturation upon β3-adrenergic activation. In Ctrl mice, mRNA of Elovl6 was induced by β3-adrenergic agonist treatment, while Scd1 expression remained the same as in vehicle condition (Figure 5A,G). Remarkably, both female and male PPARαBATKO mice fed a HFD displayed a strong induction of Scd1 mRNA level after β3-adrenergic agonist treatment (Figure 5A,G, left panels). Protein levels of SCD1 were in agreement with mRNA expression (Figure 5B). However, mRNA levels of Elovl6 were similar in the two genotypes with both conditions (Figure 5A,G, right panels). Interestingly, these changes in expression levels of genes involved in FA elongation and desaturation were associated with modified lipidomic profiles in BAT of PPARαBATKO mice. The C16:1n-7/C18:2n-6 ratio tended to be increased in BAT PL of PPARαBATKO mice after β3-adrenergic agonist treatment (Figure 5E,J), and was significantly increased in BAT TG (Figure 5C,H). The FA ratio representing the activity of SCD1, or SCD1 ratio C16:1n-7/C16:0, was consistent with gene expression, as it was increased in PPARαBATKO mice after β3-adrenergic agonist treatment in both BAT TG (Figure 5D,I, left panels) and BAT PL (Figure 5F,K, left panels) of female and male mice. Similarly to the lipogenic ratio, SCD1 ratio was reduced in male mice after CL316,243 treatment only in the control genotype for BAT TG (Figure 5I, left panel) and BAT PL (Figure 5K, left panel). The ratio representing the activity of ELOVL6 in BAT TG tended to be reduced in female PPARαBATKO mice after CL316,243 treatment (Figure 5D, right panel), and was significantly reduced for BAT PL (Figure 5F, right panel). In male animals, the ELOVL6 ratio was significantly reduced in PPARαBATKO mice after β3-adrenergic agonist treatment in both BAT TG (Figure 5I, right panel) and BAT PL (Figure 5K, right panel). Relative abundance of arachidonic acid (C20:4 n-6) was also reduced in PPARαBATKO mice after CL316,243 treatment compared to Ctrl mice (Supplementary Figure 4C and D). In summary, our data support the role of PPARα in controlling BAT FA content in TG and PL.

**Figure 5:**
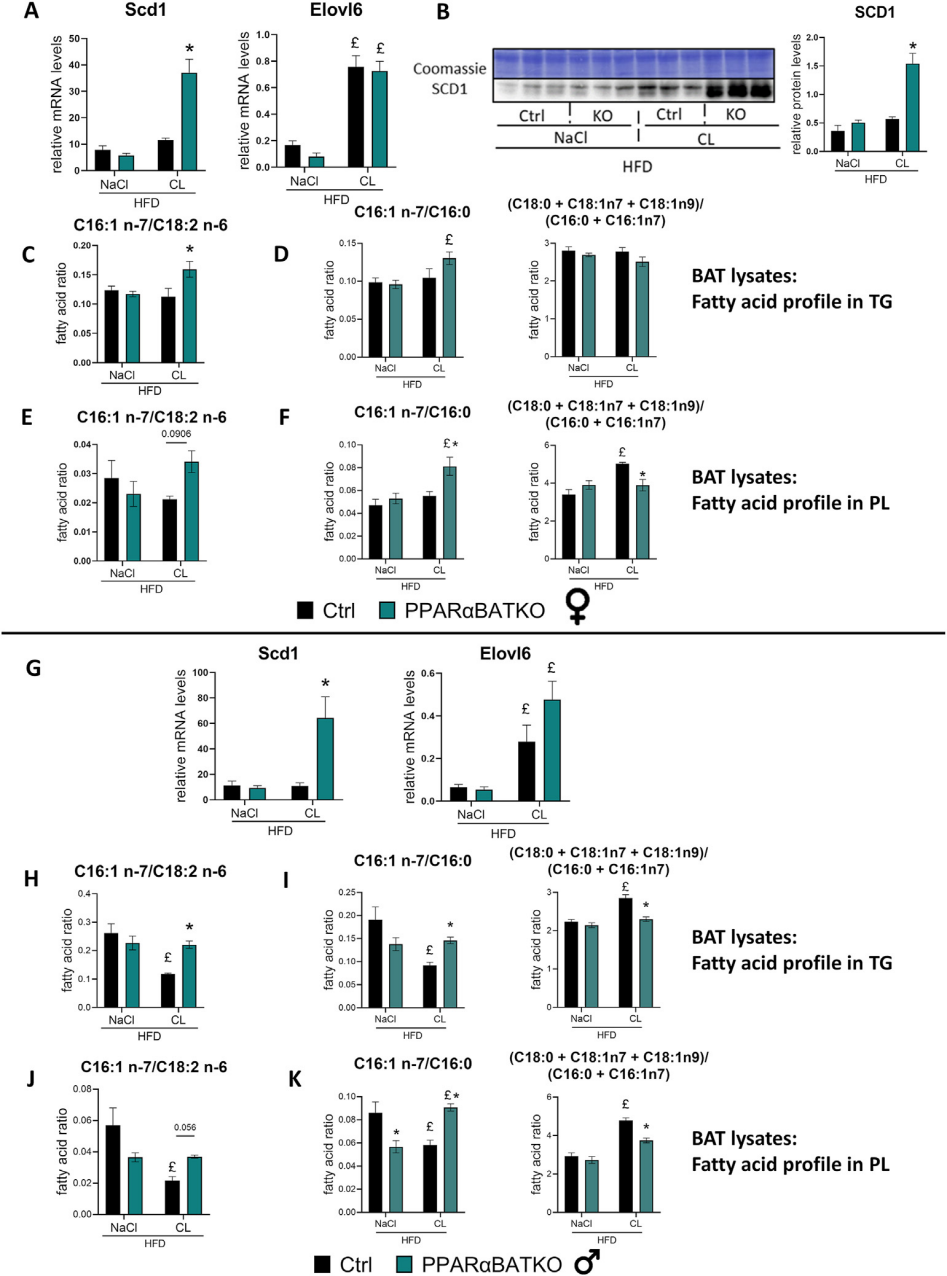
**BAT specific deletion of PPARα increases FA desaturation in BAT in HFD-induced obese mice upon β3-adrenergic stimulation.** (A, G) Relative mRNA levels of Scd1 and Elovl6 in BAT of female (A) and male (G) mice (*n* = 4–6 per group). (B) SCD1 protein content in BAT of female mice and corresponding quantification (*n* = 3 per group). (C, H) Lipogenic indexes in BAT triglycerides (TG) of female (C) and male (H) mice (*n* = 4 per group). (D, I) SCD1 and ELOVL6 ratios in BAT TG of female (D) and male (I) mice (*n* = 4 per group). (E, J) Lipogenic indexes in BAT phospholipids (PL) of female (E) and male (J) mice (*n* = 4 per group). (F, K) SCD1 and ELOVL6 ratios in BAT PL of female (F) and male (K) mice (*n* = 4 per group). Data are displayed as mean ± SEM. Statistical analysis was performed using 2-way ANOVA with Šídák’s post hoc test or Mann–Whitney test (G for Scd1). ∗*p* < 0.05, *vs* Ctrl. £*p* < 0.05, *vs* NaCl. Ctrl: control mice; KO: PPARαBATKO mice.

### 3.6 BAT specific deletion of PPARα induces glucose intolerance in mice fed a chow diet

**Figure 6:**
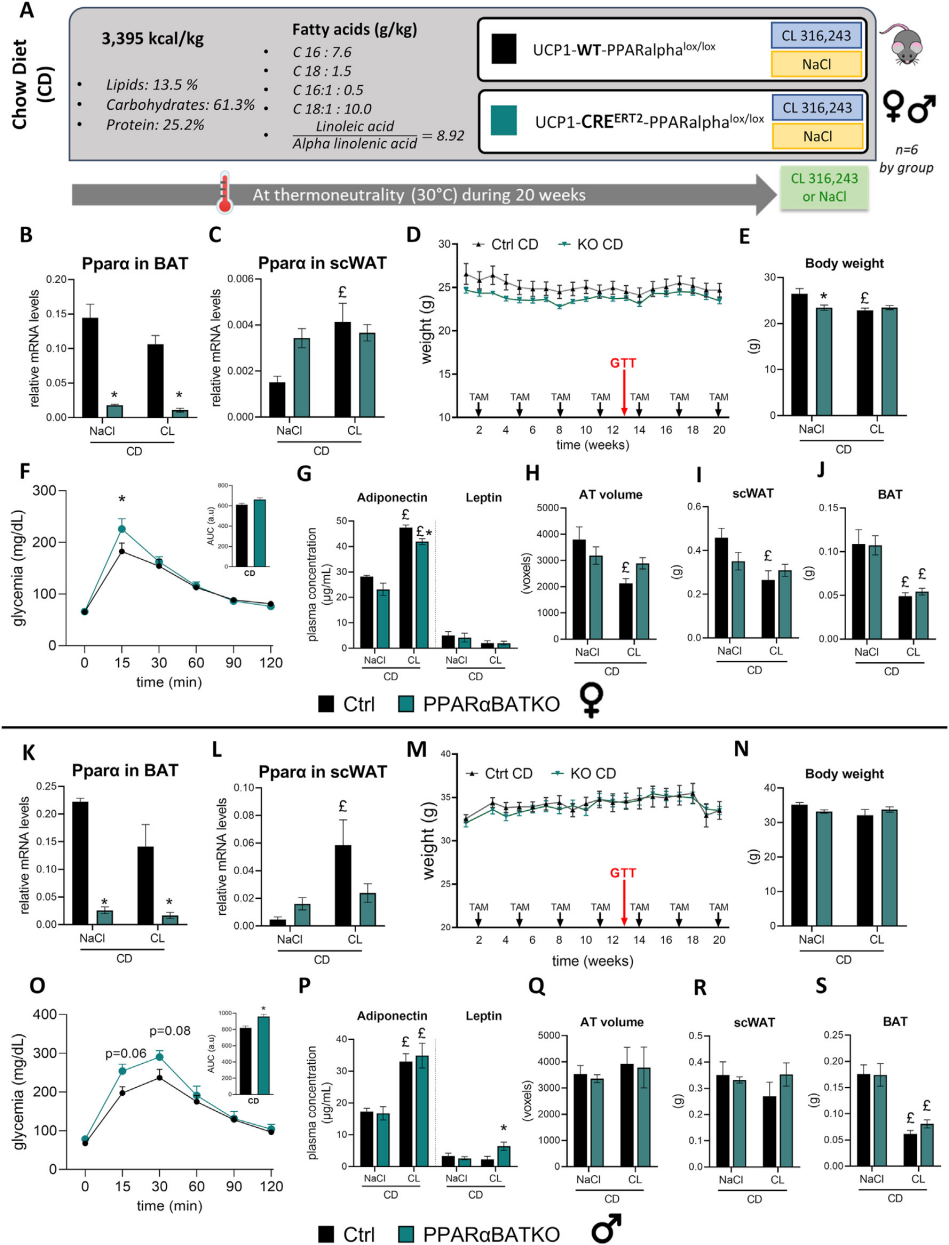
**BAT specific deletion of PPARα induces glucose intolerance in mice fed a chow diet.** (A) Experimental design. Control (UCP1-WT-PPARalphalox/lox) and PPARαBATKO (UCP1-CRE^ERT2^-PPARalphalox/lox) were housed at thermoneutrality and fed a standard chow diet (CD) for 20 weeks and daily injected with 1 mg/kg of CL316,243 or NaCl the last week of the experiment. To induce CRE^ERT2^ activity, mice were injected intraperitoneally with tamoxifen (TAM) at a dosage of 60 mg/kg the first three days of the CD, then every three weeks. (B, K) Relative mRNA levels of Pparα in brown adipose tissue (BAT) of female (B) and male (K) adult mice (*n* = 6 per group). (C, L) Relative mRNA levels of Pparα subcutaneous (scWAT) of female (C) and male (L) adult mice (*n* = 6 per group). (D, M) Weight curves of control (Ctrl) and PPARαBATKO female (D) and male (M) mice (*n* = 11–12 per group) on standard chow diet (CD) during 20 weeks. (F, O) Glucose tolerance test (GTT) after 13 weeks of CD in female (F) and male (O) mice, and corresponding area under curves (AUC) (*n* = 12 for (F), *n* = 9 for (O)). (G, P) Plasma levels of Adiponectin and Leptin in female (G) and male (P) mice (*n* = 4–6 per group). (H, Q) AT volume estimation assessed by tomography in female (H) and male (Q) adult mice (*n* = 3–5 per group). (I, R) Subcutaneous white adipose tissue (scWAT) weights in female (I) and male (R) mice (*n* = 5–6 per group). (J, S) Intrascapular brown adipose tissue (BAT) weights in female (J) and male (S) mice (*n* = 5–6 per group). Data are displayed as mean ± SEM. Statistical analysis was performed using 2-way ANOVA with Šídák’s post hoc test or Mann–Whitney test (B and K). ∗*p* < 0.05, *vs* Ctrl. £*p* < 0.05, *vs* NaCl. Ctrl: control mice; KO: PPARαBATKO mice.

We showed previously that the lack of PPARα in BAT induces the expression of DNL enzymes after β_3_-adrenergic stimulation in mice fed a HFD. Next we investigated the role of PPARα in a situation where DNL is robustly induced in BAT. To do so, Ctrl and PPARαBATKO mice were maintained at thermoneutrality (28–30 °C) and were given a CD with more than 50% of energy coming from carbohydrates for 20 weeks. The last 7 days of the experiment, mice of each genotype were divided into 2 groups (Figure 6A): half of the animals were daily injected with 1 mg/kg of CL316,243, while the other half was injected with vehicle (NaCl). PPARαBATKO female and male mice displayed, upon tamoxifen treatment, a strong inhibition of Pparα mRNA levels (Figure 6B,K) in BAT compared to Ctrl mice, whereas Pparγ and Pparδ levels were not affected (Supplementary Figure 1A and C). β3-adrenergic agonist stimulation had no significant effect on Pparα expression in BAT. Interestingly, the mRNA level of Pparα in scWAT was significantly induced by CL316,243 only in Ctrl mice, both in female and male animals (Figure 6C,L). Next, we looked at a possible organismal effect of PPARαBATKO by measuring body weight under different conditions. Interestingly, PPARαBATKO mice had the same body weight as the Ctrl mice both in female (Figure 6D) and male groups (Figure 6M). However, PPARαBATKO female mice displayed reduced body weight after vehicle injection compared to Ctrl mice, while β3-adrenergic agonist treatment reduced body weight only in Ctrl mice (Figure 6E). In male, β3-adrenergic agonist stimulation had no significant effect on body weight in both genotypes (Figure 6N). PPARαBATKO mice showed impaired glucose disposal during GTT both in female (Figure 6F) and male groups (Figure 6O). Indeed, the glycemia peak during GTT was higher in female PPARαBATKO mice, while area under the curve was significantly increased in male PPARαBATKO mice compared to Ctrl mice. After vehicle injection, circulating levels of adiponectin and leptin were the same in both genotypes. Female PPARαBATKO mice displayed reduced plasma concentration of adiponectin after β3-adrenergic agonist treatment (Figure 6G), while leptin levels were higher in male animals (Figure 6P). In female mice, the AT volume and the weight of subcutaneous fat pad were reduced by β3-adrenergic agonist treatment only in the Ctrl group (Figure 6H,I). BAT weights were similar in both genotypes treated with vehicle or β3-adrenergic agonist (Figure 6J). In male mice, β3-adrenergic agonist treatment had no effect on the volume of adipose tissue and the weight of subcutaneous fat pad in both genotypes (Figure 6Q,R). BAT weight was reduced by CL316,243 to the same extent in PPARαBATKO and control groups (Figure 6S).

### 3.7 BAT specific deletion of PPARα does not modulate BAT De Novo Lipogenesis in mice fed a chow diet but increases its fat storage

**Figure 7:**
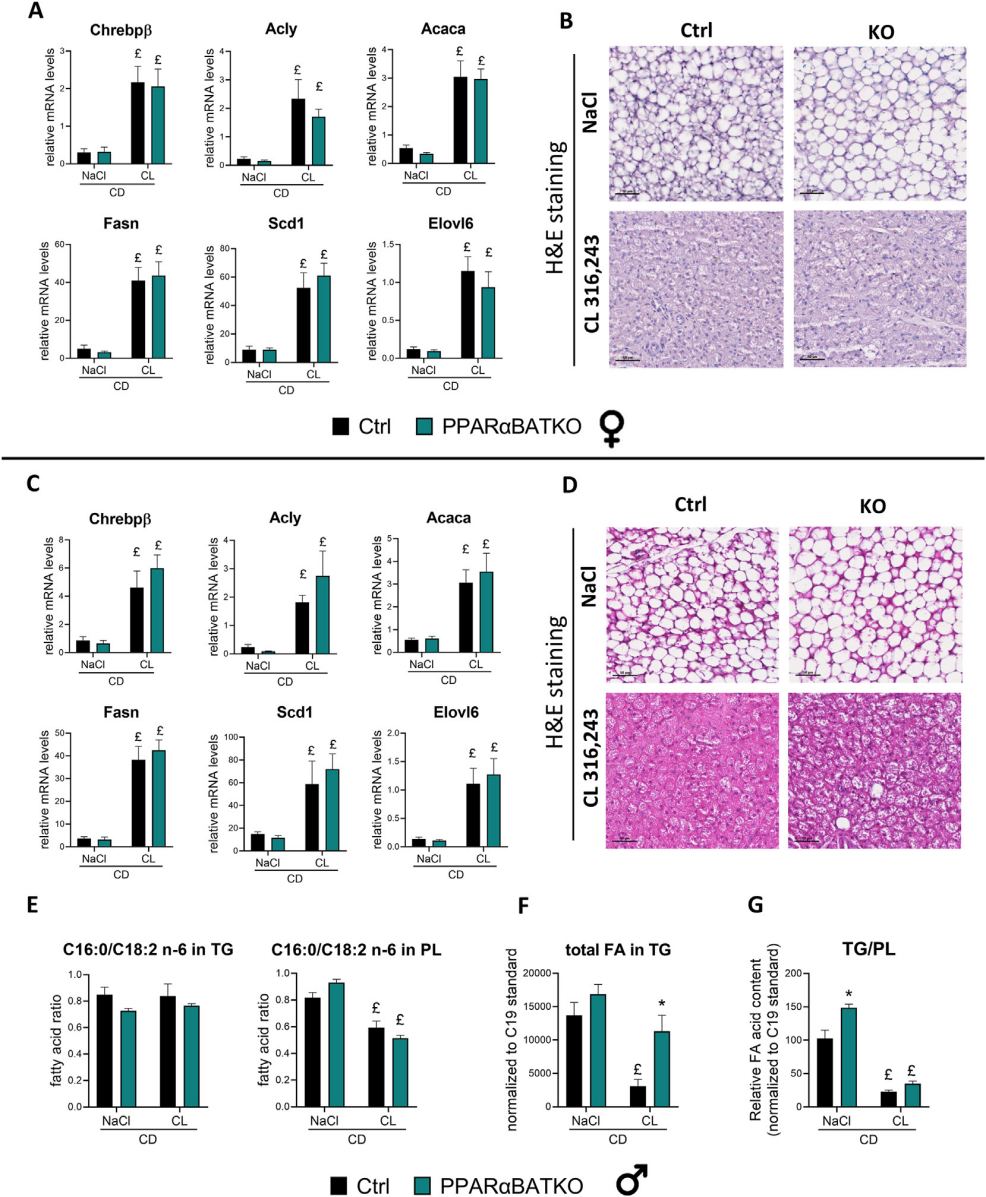
**BAT specific deletion of PPARα does not modulate BAT De Novo Lipogenesis in mice fed a chow diet but increases fat storage.** (A, C) Relative mRNA levels of De Novo Lipogenesis (DNL) genes in brown adipose tissue (BAT) of female (A) and male (C) mice (*n* = 6 per group). (B, D) Representative H&E staining of BAT in female (B) and male (D) mice fed a HFD (scale = 50 μm). (E) Lipogenic index in in BAT triglycerides (TG) and phospholipids (PL) of male mice (*n* = 4 per group). (F) Relative fatty acid content in BAT triglycerides compared to internal standard C19 of male mice (*n* = 4 per group). (G) Relative triglycerides to phospholipids fatty acid content in BAT of male mice (*n* = 4 per group). Data are displayed as mean ± SEM. Statistical analysis was performed using 2-way ANOVA with Šídák’s post hoc test or Mann–Whitney test (A for Elovl6, C for Chrebpb, Acly, Acaca, Fasn and Scd1). ∗*p* < 0.05, *vs* Ctrl. £*p* < 0.05, *vs* NaCl. Ctrl: control mice; KO: PPARαBATKO mice.

The consequences of the deletion of PPARα in BAT depend on the metabolic context, especially the diet. We showed that glucose tolerance was slightly altered only in PPARαBATKO mice fed a CD. We also demonstrated that DNL was highly induced in PPARαBATKO mice under HFD and treated with β3-adrenergic agonist. DNL is essential for BAT homeostasis and cold response. In mice fed a CD, the mRNAs of ChREBPβ and its associated genes involved in DNL were induced to the same extent in the PPARαBATKO group compared to the control group in both sexes (Figure 7A,C). The expression of genes involved in elongation and desaturation was the same in both genotypes (Figure 7A,C). The expression of other DNL modulators, Chrebpalpha and Srebp1, remained unaltered (Supplementary Figure 1D and E). In agreement with the gene expression, the lipogenic index was not modified in PPARαBATKO mice, neither in BAT TG, nor in BAT PL (Figure 7E). However, histological analyses revealed that PPARαBATKO mice seem to display larger BAT lipid droplets in the vehicle condition (Figure 7B,D). Fat storage seemed to be increased, at least in male PPARαBATKO mice, as total FA content in TG was higher compared to Ctrl mice upon β3-adrenergic agonist treatment (Figure 7F). In male mice, the relative content of FA in TG compared to FA in PL tended to be increased in PPARαBATKO mice after CL316,243 treatment (Figure 7G). This ratio was significantly higher in PPARαBATKO after vehicle injections (Figure 7G).

## 4 Discussion

While the role of PPARα in the liver is well established [17,37], its function in BAT remains unclear. Hepatic PPARα is required for optimal BAT activation in response to β3-adrenergic stimulation. Indeed, mice lacking PPARα only in the liver are cold intolerant and display reduced BAT activation by CL316,243 injection [37]. Previous studies focused mostly on the role of adipose PPARα in thermogenesis and fatty acid oxidation. Products from lipolysis can activate PPARα and PPARδ in BAT providing a mechanism for matching oxidative capacity to substrate supply [38]. However, adipose PPARα is dispensable to maintain body temperature upon cold exposure [13,19,21]. These data were obtained in mice fed a standard CD and with global knock-out models, which implies that Pparα expression was suppressed not only in adipocytes, but also in precursors and other stromal cells. A study from Lasar et al. based on the same model as the one used in our study showed that the deletion of PPARα under the control of an inducible Ucp1 promotor does not affect the oxygen consumption, neither at room temperature, nor at 4 °C [13,19,21]. This last study was conducted with mice fed a CD. In our work, we took advantage of this BAT-specific deletion model (PPARαBATKO) to investigate the role of PPARα in a unique experimental condition, i.e. housing at thermoneutrality with an obesogenic diet followed by β3-adrenergic receptor stimulation, which has never been performed, to the best of our knowledge. Regarding BAT thermogenesis, we showed that the absence of PPARα in BAT only modestly decreased UCP1 levels after β3-adrenergic agonist treatment. This effect was observed in female mice fed with HFD or with CD, but not in male mice, regardless the diet. These data suggest that BAT PPARα seems to be dispensable for optimal UCP1-dependent BAT thermogenesis. As UCP1 is a mitochondrial protein, we also investigated mitochondrial dynamics and biogenesis. We found that in PPARαBATKO female mice fed a HFD and treated with a β3-adrenergic agonist treatment, the amount of DRP1 protein, a mitochondrial protein highly expressed in BAT and associated with WAT browning [39], is markedly reduced. In adipose tissue upon β-adrenergic stimulation, DRP1 facilitates lipid droplet budding from the ER, and hence, new cytosolic lipid droplet formation. Our data are also consistent with the fact that Adipo-Drp1flx/flx mice exhibit adipocytes with larger sizes and form less multilocular structures upon cold exposure [40]. DRP1 is also essential for mitochondrial fission which facilitates mitophagy for the recycling of damaged mitochondria. Considering this, PPARαBATKO female mice might have altered mitophagy. However, no difference was observed between both genotypes for OXPHOS complexes. Nevertheless, PPARαBATKO female mice display not only lower relative mitochondrial DNA content when treated with CL316,243, compared to Ctrl mice, but also lower mRNA levels of Ppargc1α. Taken together, these data suggest an impaired mitochondrial biogenesis.

It is known for several years that lipogenesis is a process highly modulated by metabolic challenges. In recent years, there has been a growing interest in BAT lipogenesis. In BAT of mice fed a CD, acute CL316,243 injection upregulates TG re-esterification, but inhibits DNL [41]. However, DNL-associated genes are among the most upregulated genes by chronic activation and mild cold in BAT. A recent study confirmed that increased DNL after cold exposure was BAT-specific [42]. The situation is different in mice challenged with a HFD. In the context of obesity, adipose tissue function is dysregulated, leading to increased basal lipolysis and impaired lipid incorporation [43,44]. As a consequence, circulating free FAs and hepatic DNL are increased, while lipogenesis is decreased in adipose tissue [45]. Mice maintained on HFD exhibit decreased levels of ChREBPβ, as well as of Acly, Acc, and Fasn in both WAT and BAT [34]. When energy intakes are higher than energy expenditure, BAT undergoes a “whitening” process leading to increased lipid deposition. Nutrient availability is high, especially at 30 °C, so that the DNL is not required to maintain energy homeostasis. Therefore, the DNL should not be induced upon HFD in BAT, which is the case in Ctrl mice in our study. However, in PPARαBATKO mice fed a HFD and treated with CL316,243, we found that ChREBPβ and its DNL-target genes are still induced and to the same extent as that observed in Ctrl mice on CD. Thus, PPARα appears to be an energy sensor essential for both FA anabolism and catabolism. PPARα was previously shown to be involved in the regulation of lipogenesis as adipocyte-specific deletion of PPARα increased SREBP1-lipogenesis in WAT [22]. In WAT, deletion of the target genes FASN, ACC1 or both enzymes, induces browning of white adipocytes and Scd1 controls de novo beige fat biogenesis [46,47]. In our study, the phenotype observed in PPARαBATKO mice fed a HFD is broadly speaking quite similar to the one obtained in mice overexpressing ChREBPβ specifically in BAT [48]. Indeed, these mice display increased DNL and have morphological changes in BAT with increased lipid deposition, associated with reduced expression of thermogenic markers and mitochondrial content as well as cold intolerance. Sanchez-Gurmaches et al. published that AKT2 drives cold-related lipogenesis induced in BAT by stimulating ChREBP activity [34]. They also reported that AKT2 levels and activity were reduced by an obesogenic diet. In our study, AKT2 was upregulated in PPARαBATKO mice on HFD. PPARαBATKO mice fed a HFD seem to lack metabolic sensors to maintain lipid homeostasis. As a result, the morphology of BAT is changed markedly, at least in female mice. To sum up, mice overexpressing ChREBPβ specifically in BAT or lacking PPARα in BAT exhibit the characteristics of BAT whitening.

Since the DNL was not previously investigated in PPARαBATKO mice under CD [13], we studied the DNL in these mice, using the same experimental protocol as the one used for HFD-fed PPARαBATKO mice. We found that mRNAs of genes involved in DNL were similarly induced upon CL316,243 treatment in both Ctrl and PPARαBATKO groups, regardless of sex. However, glucose tolerance was impaired, as well as fat storage in BAT, which was associated with larger lipid droplets. These results indicate that BAT specific deletion of PPARα might impair systemic glucose tolerance, which might be due, at least in part, to a whitening of BAT under CD. In comparison, under HFD, glucose intolerance was not further impaired in PPARαBATKO mice compared to Ctrl mice, but increased fat storage was still observed. Moreover, ChREBPβ was strongly induced and this was associated with increased expression of enzymes involved in DNL. Iizuka et al. previously showed in differentiated brown adipocytes that ChREBPβ mRNA levels were induced and PPARα mRNA levels were suppressed by glucose, whereas PPARα suppressed glucose induction of ChREBPβ target gene expression [49]. Therefore, under CD, composed of high amounts of carbohydrates, the deletion of PPARα might have no impact on ChREBPβ-mediated DNL which was similarly induced upon CL316,243 treatment in both genotypes. In contrast, under HFD, if the presence of high amounts of lipids does not seem to impair the balance between ChREBPβ and PPARα in Ctrl mice, in PPARαBATKO mice, PPARα-induced ChREBPβ repression is lost, leading to highly increased expression of ChREBPβ and its target genes. Together this eventuates in deregulation of lipid metabolism in BAT.

It is generally accepted now that obesity is associated with fibro-inflammation in WAT restricting healthy plasticity of adipose tissue [50]. However, fibrotic remodeling of BAT remains poorly clarified [51]. Here we report that obese female PPARαBATKO mice exhibit larger lipid droplets in BAT as well as more Picro-Sirius staining after a β3-adrenergic stimulation. This suggests that PPARαBATKO mice have more fibrosis in BAT which might aggravate adipose dysfunction.

In our study, we show that the absence of PPARα in BAT increased the synthesis of FAs by FASN in mice fed a HFD and treated with CL316,243. Moreover, expression of SCD1 was also increased while the levels of ELOVL6 remained the same between both genotypes. As a result, PPARαBATKO mice displayed impaired FA profiles in TG and PL, especially after β3-adrenergic stimulation. In agreement with increased levels of DNL genes from Acly to Scd1, relative abundances of palmitoleate in TG and PL were increased in PPARαBATKO mice. Monounsaturated FAs, especially palmitoleate, can down-regulate Ucp1 expression via attenuation of βAR-cAMP-CREB pathway through increased PDE activity [52]. Stimulations such as cold exposure or β3-adrenergic agonists also increase the expression and activity of enzymes involved in lipid oxidation. Altogether, these changes in lipid synthesis might lead to modifications in the composition of cell and organelles membranes and thus in their physical properties. They could also lead to different relative amounts of bioactive lipids produced from PL, such as oxylipins, which are important signaling molecules in health and disease. For instance, metabolites derived from arachidonic acid, such as prostaglandin E2 and prostacyclin promote the formation of beige adipocytes [53,54]. Alterations in the synthesis of these molecules could have a significant impact on adipose tissue homeostasis.

### 4.1 Limitations of the study

Our study unveils a novel role for BAT PPARα in the regulation of energy homeostasis. However, we did not measure parameters such as temperature or oxygen consumption to verify whether energy expenditure was indeed affected. Moreover, the diet used in the present study was a HFD with 60% of energy coming from lipids. One can question whether the absence of PPARα in BAT would have the same consequences with another nutritional challenge, such as a high fructose diet or high protein diet. Upregulation of DNL in PPARαBATKO mice was observed after β3-adrenergic agonist treatment, and was not investigated in response to cold exposure (4 °C). However, histologic analysis revealed that PPARαBATKO mice housed at 22 °C, conditions in which BAT is activated, displayed the same BAT alterations as those housed at thermoneutrality (Supplementary Figure 5).

## 5 Conclusion

Our findings demonstrate an important novel role for PPARα in BAT lipogenesis. This unexpected function has been identified because of the unique experimental protocol used in this study which combined housing at thermoneutrality and a HFD, followed by a β3-adrenergic agonist treatment. PPARα specifically regulates ChREBPβ-mediated De Novo Lipogenesis. Hence, PPARα appears to be essential to maintain BAT lipid homeostasis. The upregulation of DNL after a combination of a HFD and β3-adrenergic stimulation alters BAT morphology, leading to increased BAT whitening, at least in female mice, and modification of FA profiles in TG and PL in both male and female mice. The crosstalk between ChREBPβ and PPARα in brown adipocytes might be of particular importance in the dysregulation of lipid metabolism in obesity.

## CRediT authorship contribution statement

**Pierre-Louis Batrow:** Writing – review & editing, Writing – original draft, Methodology, Investigation, Formal analysis, Data curation, Conceptualization. **Sylvie Caspar-Bauguil:** Writing – review & editing, Investigation, Formal analysis. **Nathalie Rochet:** Methodology, Investigation, Formal analysis, Data curation. **Nadine Gautier:** Writing – review & editing, Methodology, Investigation, Formal analysis, Data curation. **Anne-Sophie Rousseau:** Writing – review & editing, Formal analysis. **Marielle Maret:** Writing – review & editing, Data curation. **Samah Rekima:** Writing – review & editing, Data curation. **Etienne Mouisel:** Writing – review & editing, Methodology. **Emmanuel Van Obberghen:** Writing – review & editing. **Christian H. Roux:** Writing – review & editing. **Hervé Guillou:** Writing – review & editing, Formal analysis. **Catherine Postic:** Writing – review & editing, Formal analysis. **Christian Wolfrum:** Writing – review & editing, Resources. **Dominique Langin:** Writing – review & editing, Formal analysis. **Ez-Zoubir Amri:** Writing – review & editing, Writing – original draft, Supervision, Project administration, Methodology, Funding acquisition, Formal analysis, Data curation, Conceptualization. **Isabelle Mothe-Satney:** Writing – review & editing, Writing – original draft, Supervision, Project administration, Methodology, Investigation, Formal analysis, Conceptualization.

## Declaration of competing interest

The authors declare that they have no known competing financial interests or personal relationships that could have appeared to influence the work reported in this paper.

## Figures and Tables

**figs1:**
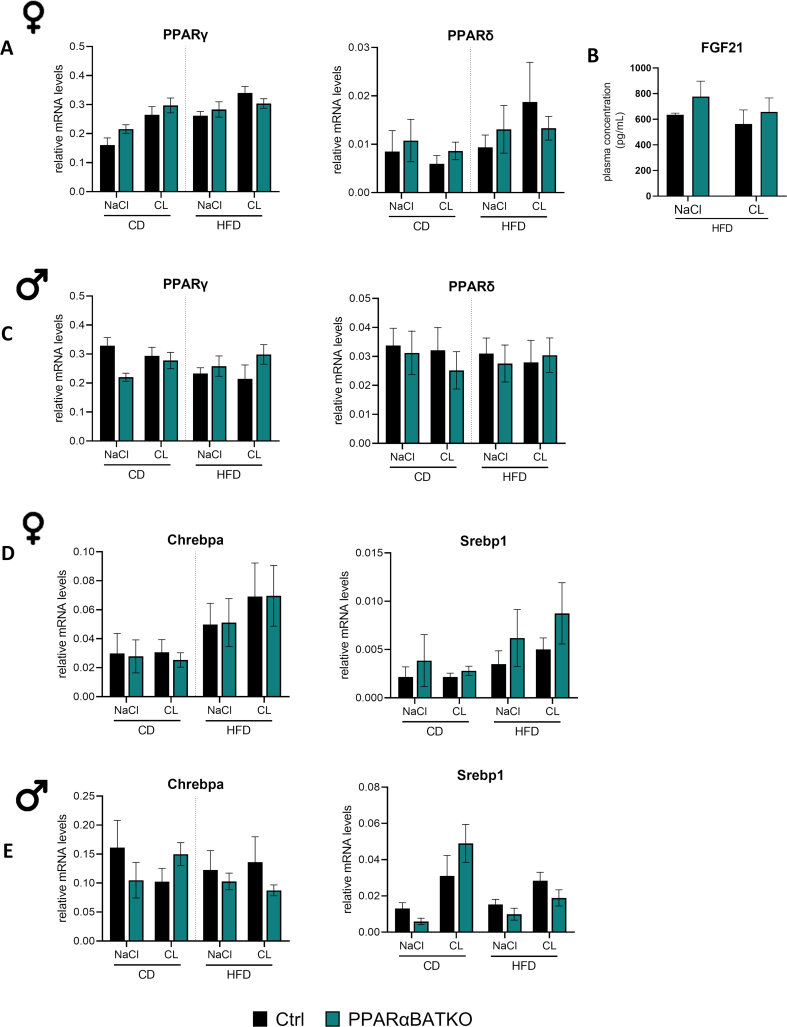
Supplementary F1, related to Figures 1, 4 and 7. (A) Relative mRNA levels of Pparγ and Pparδ in brown adipose tissue (BAT) of female adult mice fed a standard chow diet (CD) or high fat diet (HFD) (*n* = 5–6 per group). (B) Plasma level of FGF21 in female mice fed a HFD (*n* = 4 per group). (C) Relative mRNA levels of Pparγ and Pparδ in BAT of male adult mice (*n* = 5–6 per group). (D) Relative mRNA levels of Chrebpa and Srebp1 in BAT of female adult mice (*n* = 5–6 per group). (E) Relative mRNA levels of Chrebpa and Srebp1 in BAT of male adult mice (*n* = 5–6 per group). Data are displayed as mean ± SEM. Statistical analysis was performed using 2-way ANOVA with Šídák’s post hoc test. NaCl: vehicle; CL: CL316,243 (**β3-adrenergic agonist**). Ctrl: control mice; KO: PPARαBATKO mice.

**figs2:**
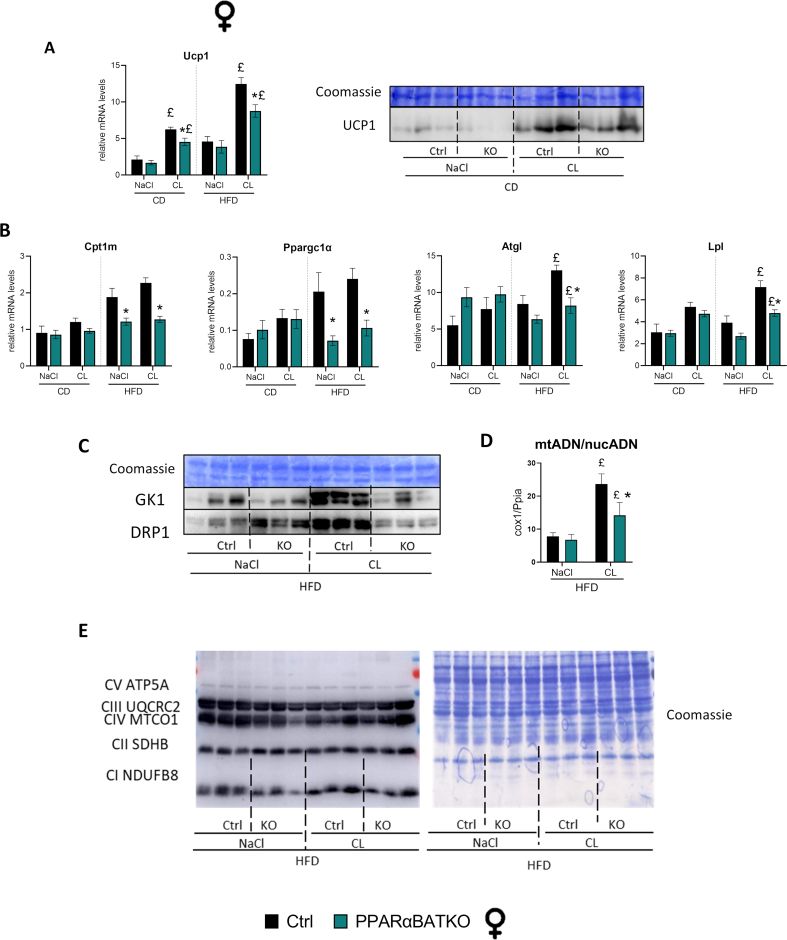
Supplementary F2, related to Figure 3. (A) Relative mRNA levels of Ucp1 in brown adipose tissue (BAT) of female mice fed a standard chow diet (CD) or a high fat diet (HFD) (*n* = 4–6 per group); UCP1 protein content in BAT of female mice fed a standard chow diet. (B) Relative mRNA levels of Cpt1m, Ppargc1**α**, Atgl and Lpl in BAT of female adult mice fed a standard chow diet or a high fat diet (*n* = 5–6 per group). (C) Western bots of DRP1 and GK1 protein in BAT of female mice fed a high fat diet (*n* = 3 per group). (D) Relative mitochondrial DNA content in BAT of female mice fed a high fat diet (*n* = 4–6 per group). (E) OXPHOS protein content in BAT of female mice fed a high fat diet (*n* = 3 per group). Data are displayed as mean ± SEM. Statistical analysis was performed using 2-way ANOVA with Šídák’s post hoc test. ∗*p* < 0.05, *vs* Ctrl. £ *p* < 0.05, *vs* NaCl. NaCl: vehicle; CL: CL316,243 (**β3-adrenergic agonist**). Ctrl: control mice; KO: PPARαBATKO mice, CD: chow diet.

**figs3:**
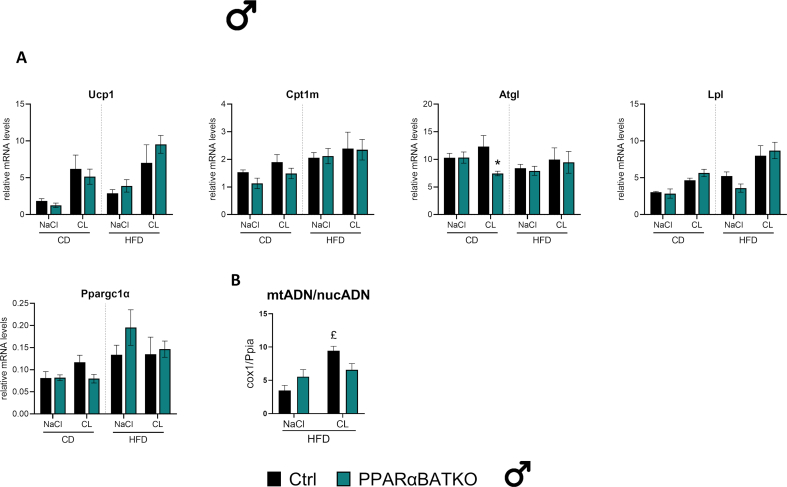
Supplementary F3, related to Figure 3. (A) Relative mRNA levels of Ucp1, Cpt1m, Atgl, Lpl and Ppargc1α in BAT of male adult mice fed a standard chow diet or a high fat diet (*n* = 5–6 per group). (B) Relative mitochondrial DNA content in BAT of female mice fed a high fat diet (*n* = 3 per group). Data are displayed as mean ± SEM. Statistical analysis was performed using 2-way ANOVA with Šídák’s post hoc test. ∗*p* < 0.05, *vs* Ctrl. £ *p* < 0.05, *vs* NaCl. NaCl: vehicle; CL: CL316,243 (**β3-adrenergic agonist**). Ctrl: control mice; KO: PPARαBATKO mice, CD: chow diet.

**figs4:**
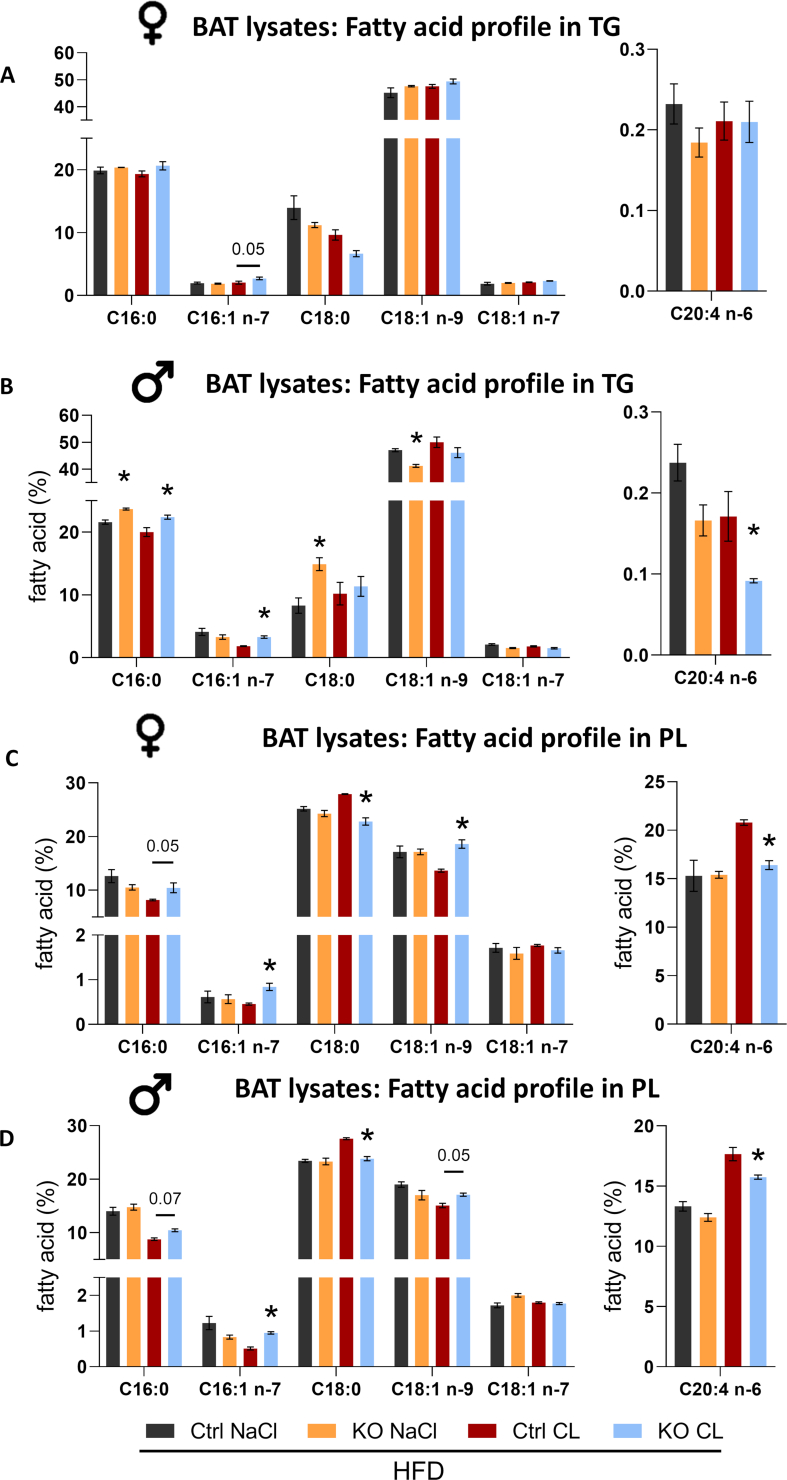
Supplementary F4, related to Figures 4 and 5. (A, B) Triglyceride fatty acid profile in BAT of female (A) and male (B) mice fed a HFD and challenged with CL316,243 (CL) or vehicle (NaCl) (*n* = 4 per group). (C, D) Phospholipid fatty acid profile in BAT of female (C) and male (D) mice fed a HFD and challenged with CL316,243 (CL) or vehicle (NaCl) (*n* = 4 per group). Data are displayed as mean ± SEM. Statistical analysis was performed using 2-way ANOVA with Šídák’s post hoc test. ∗*p* < 0.05, *vs* Ctrl. Ctrl: control mice; KO: PPARαBATKO mice.

**figs5:**
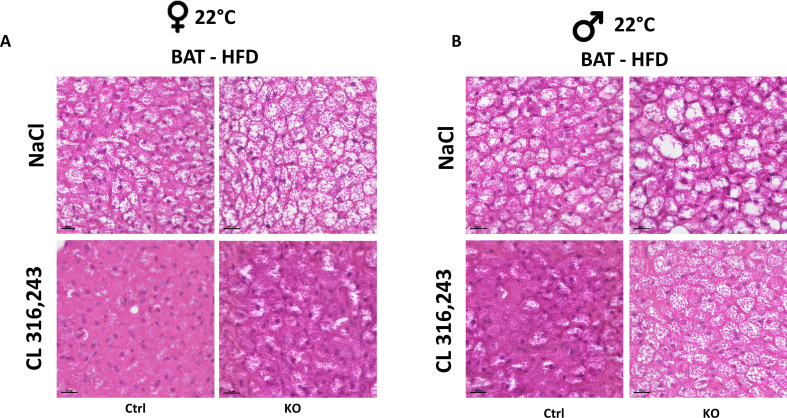
Supplementary F5, related to Figure 3. (A, B) Representative H&E staining of BAT in female (A) and male (B) mice fed a HFD and housed at 22 °C (scale = 20 μm).

## Data Availability

Data will be made available on request.
