# Supplementary material for: Deletion of PPARα in mouse brown adipocytes increases their De Novo Lipogenesis
Source: Mol Metab. 2025 Jun 10;98:102184. doi: 10.1016/j.molmet.2025.102184 (PMC12221382; doi:10.1016/j.molmet.2025.102184)
Supplement: Multimedia component 1 [file mmc1.docx]

**Supplementary Table 1**

| **Gene names** | **Forward primer** | **Reverse primer** |
| --- | --- | --- |
| COX1 (mitochondrial DNA) | ACTATACTACTACTAACAGACCG | GGTTCTTTTTTTCCGGAGTA |
| PPIA (nuclear DNA) | ACACGCCATAATGGCACTGG | CAGTCTTGGCAGTGCAGAT |
| Ppara | AGTTCACGCATGTGAAGGCTG | TGTTCCGGTTCTTCTTCTGAATC |
| Pparg | CATAAAGTCCTTCCCGCTGA | GAAACTGGCACCCTTGAAAA |
| Ppard | AGATGGTGGCAGAGCTATGACC | TCCTCCTGTGGCTGTTCC |
| Adiponectin | GGAGAGAAAGGAGATGCAGGT | CTTTCCTGCCAGGGGTTC |
| Leptin | CAGGATCAATGACATTTCACACA | GCTGGTGAGGACCTGTTGAT |
| Ucp1 | CACCTTCCCGCTGGACACT | CCTGGCCTTCACCTTGGAT |
| Cpt1m | TGCCTTTACATCGTCTCCAA | GGCTCCAGGGTTCAGAAAGT |
| Atgl | CAAGGGGTGCGCTCTGTGGATGG | AGGCGGTAGAGATTGCGAAGGTTG |
| Lpl | TTATCCCAATGGAGGCACTTT | CACGTCTCCGAGTCCTCTCTC |
| Gk | CAAATGCAAGCAGGACGATG | AGGCCCCAGCTTTCATTAGG |
| Ppargc1a | GAAAGGGCCAAACAGAGAGA | GTAAATCACACGGCGCTCTT |
| Letmd1 | CTACCCACATTGCTGACGAC | GGGACAGCTCGGGTTCTT |
| Mlxipl (ChREBPα) | CGACACTCACCCACCTCTTC | TTGTTCAGCCGGATCTTGTC |
| Mlxipl (ChREBPβ) | TCTGCAGATCGCGTGGAG | CTTGTCCCGGCATAGCAAC |
| Srebf1 | GCATGCCATGGGCAAGTAC | TGTTGCCATGGAGATAGCATCT |
| Acly | TGTGCTCGGGCTGGGAAGGAC | GTGGCGGGGAAGTGCTGTTTGA |
| Acaca | GAAGGCATCCCACGCATCT | GCGTATTTCTTCTGCAAGTCCAA |
| Acacb | CTCGAAGCCGCTCACCAA | GAAAGGGACTCCTCGCTTGAA |
| Scd1 | CCTTCCCCTTCGACT | GCCATGCAGTCGATG |
| Fasn | GCTGCGGAAACTTCAGGAAAT | AGAGACGTGTCACTCCTGGACTT |
| Elovl6 | TCAGCAAAGCACCCGAAC | AGCGACCATGTCTTTGTAGGAG |
| Cox2 | TGCTGGTGGAAAAACCTCGT | AAAACCCACTTCGCCTCCAA |
| Col1a1 | GCTCCTCTTAGGGGCCACT | CCACGTCTCACCATTGGGG |
| Col3a1 | CTGTAACATGGAAACTGGGGAAA | CCATAGCTGAACTGAAAACCACC |
| Col6a1 | CTGCTGCTACAAGCCTGCT | CCCCATAAGGTTTCAGCCTCA |
| 36b4 | CTTTATCAGCTGCACATCACTCAGA | TCCAGGCTTTGGGCATCA |
