# Supplementary material for: Deletion of PPARα in mouse brown adipocytes increases their De Novo Lipogenesis
Source: Mol Metab. 2025 Jun 10;98:102184. doi: 10.1016/j.molmet.2025.102184 (PMC12221382; doi:10.1016/j.molmet.2025.102184)
Supplement: Multimedia component 2 [file mmc2.docx]

**Supplementary Table 2**

| REAGENT or RESOURCE | SOURCE | IDENTIFIER |
| --- | --- | --- |
| Antibodies | | |
| UCP1 | Abcam | Ab10983 |
| GK1 | Santa Cruz | sc-393555 |
| DRP1 | CST | #5391 |
| OXPHOS | Abcam | ab110413 |
| ACC | CST | #3662 |
| Tubulin | CST | #3873 |
| Phospho-ACC1 (Ser79)[Ser80] | Affinity | AF3421 |
| FASN | ABclonal | A19050 |
| ACLY | Santa Cruz | sc-517267 |
| SCD | ABclonal | A16429 |
| Pan-ACTIN | CST | #8456 |
| AKT2 | CST | #2964 |
| PKM2 | ABclonal | A20991 |
| Anti-Mouse IgG (H+L), HRP Conjugate | Promega | W402B |
| Anti-Rabbit IgG (H+L), HRP Conjugate | Promega | W401B |
| Chemicals, peptides, and recombinant proteins | | |
| CL316,243 |  |  |
| Tamoxifen | Sigma | T5648-5G |
| sunflower seed oil | Sigma | S5007 |
| Critical commercial assays | | |
| Fibroblast Growth Factor 21 Mouse/Rat ELISA | Biovendor | RD291108200R |
| Adiponectin (mouse) ELISA | Bertin Bioreagent | A05187 |
| Leptin (mouse, rat) ELISA | Bertin Bioreagent | A05176 |
| Experimental models: Organisms/strains | | |
| Mouse: C57BL/6N UCP1-CRE^ERT2^ | Christian Wolfrum |  |
| Mouse: C57BL/6N PPARalpha^lox/lox^ | Christian Wolfrum |  |
| Software and algorithms | | |
| QuPath v5.1 | QuPath Software | https://qupath.github.io/ |
| GraphPad Prism v9.1.1 | GraphPad Software Inc. | https://www.graphpad.com/ |
| Fiji | ImageJ | https://imagej.net/ |
